# Exo-Metabolome of *Pseudovibrio* sp. FO-BEG1 Analyzed by Ultra-High Resolution Mass Spectrometry and the Effect of Phosphate Limitation

**DOI:** 10.1371/journal.pone.0096038

**Published:** 2014-05-02

**Authors:** Stefano Romano, Thorsten Dittmar, Vladimir Bondarev, Ralf J. M. Weber, Mark R. Viant, Heide N. Schulz-Vogt

**Affiliations:** 1 Max Planck Institute for Marine Microbiology, Bremen, Germany; 2 Research Group for Marine Geochemistry, Institute for Chemistry and Biology of the Marine Environment (ICBM), University of Oldenburg, Oldenburg, Germany; 3 School of Biosciences, University of Birmingham, Birmingham, United Kingdom; 4 Department of Biological Oceanography, Leibniz-Institute for Baltic Sea Research Warnemuende (IOW), Rostock, Germany; University of New South Wales, Australia

## Abstract

Oceanic dissolved organic matter (DOM) is an assemblage of reduced carbon compounds, which results from biotic and abiotic processes. The biotic processes consist in either release or uptake of specific molecules by marine organisms. Heterotrophic bacteria have been mostly considered to influence the DOM composition by preferential uptake of certain compounds. However, they also secrete a variety of molecules depending on physiological state, environmental and growth conditions, but so far the full set of compounds secreted by these bacteria has never been investigated. In this study, we analyzed the exo-metabolome, metabolites secreted into the environment, of the heterotrophic marine bacterium *Pseudovibrio* sp. FO-BEG1 via ultra-high resolution mass spectrometry, comparing phosphate limited with phosphate surplus growth conditions. Bacteria belonging to the *Pseudovibrio* genus have been isolated worldwide, mainly from marine invertebrates and were described as metabolically versatile *Alphaproteobacteria*. We show that the exo-metabolome is unexpectedly large and diverse, consisting of hundreds of compounds that differ by their molecular formulae. It is characterized by a dynamic recycling of molecules, and it is drastically affected by the physiological state of the strain. Moreover, we show that phosphate limitation greatly influences both the amount and the composition of the secreted molecules. By assigning the detected masses to general chemical categories, we observed that under phosphate surplus conditions the secreted molecules were mainly peptides and highly unsaturated compounds. In contrast, under phosphate limitation the composition of the exo-metabolome changed during bacterial growth, showing an increase in highly unsaturated, phenolic, and polyphenolic compounds. Finally, we annotated the detected masses using multiple metabolite databases. These analyses suggested the presence of several masses analogue to masses of known bioactive compounds. However, the annotation was successful only for a minor part of the detected molecules, underlining the current gap in knowledge concerning the biosynthetic ability of marine heterotrophic bacteria.

## Introduction

Microorganisms dynamically interact with their environment, they are influenced by its composition and, in turn, they influence its composition. This reciprocity has an effect on bacterial gene expression, protein synthesis, and metabolite uptake and production. In the ocean the dissolved organic matter (DOM), which consists of a collection of reduced carbon compounds often containing heteroatoms (e.g. N, P, S), is the result of these interconnected processes. Photosynthetic and non-photosynthetic bacteria can release metabolites into the environment according to their physiological state [1]. Examples are compounds secreted for nutrient acquisition (e.g. siderophores), for communication (e.g. homoserine lactones), and for interspecies competition (e.g. antibiotics). Several studies have investigated the effect of the activity of photosynthetic bacteria on DOM composition (reviewed in [1] and [2]), whereas the composition of the DOM produced by heterotrophic bacteria is almost unknown. Special attention has been paid to metabolites of biotechnological interest, but little is known about the full suite of compounds produced by bacteria under different nutrient regimes and growth phases, resulting in a general lack of information on the influence that the metabolism of marine heterotrophic bacteria has on oceanic DOM composition [2].

Metabolomics is the field of science that aims to characterize and quantify metabolites, or low molecular weight molecules, originating from cellular activity under a given set of physiological conditions. This collection of metabolites is termed the metabolome [3], which can be partitioned into the so called endo-metabolome (all intracellular metabolites) and the exo-metabolome (all extracellular metabolites) [4]–[6]. Metabolomics is a “downstream” approach and reflects the final response of cells to specific environmental conditions and it completes and integrates the associated techniques of proteomics and transcriptomics [3]. Microbial metabolomic studies have already been performed for different purposes, e.g. to elucidate metabolic pathways, to investigate the response of bacterial metabolism to environmental stresses, to support bacterial identification, and to diagnose bacterial infections [7]–[13]. Such studies have the potential to provide new insights into the composition of the metabolites secreted by marine heterotrophic bacteria and into their influence on the oceanic DOM composition.

Among the different analytical techniques, high resolution accurate mass (HRAM) mass spectrometry has acquired a predominant position in metabolomic studies [14]. Among others, Fourier transform ion cyclotron resonance mass spectrometry (FT-ICR-MS) is emerging as the most promising technology since it provides accurate mass measurement with ppm or sub-ppm error. It allows to obtain ultra-high resolved profiles with thousands of accurate masses, which in principle can be transformed into real elementary composition [15]–[18]. Therefore, it permits high-throughput screening of intracellular and extracellular metabolites providing overall information on bacterial metabolism.

This technique was successfully employed to analyze the variation in the endo-metabolome during bacterial growth, in studies of metabolic diversity among different ecotypes and in analyzing bacterial response to stress conditions [19]–[22]. However, studies that analyze the bacterial exo-metabolome during growth and in response to nutrient limitation are missing. In the present manuscript we report a detailed analysis of the exo-metabolome of strain FO-BEG1, which belongs to the genus *Pseudovibrio*. These are heterotrophic *Alphaproteobacteria* distributed worldwide and they have been detected especially in association with marine invertebrates [23], [24]. Bacteria belonging to this genus have often been shown to produce bioactive secondary metabolites, and they are considered a potential source of new molecules of medical interest [23], [25]–[27].

We investigated the composition of the secreted metabolites during bacterial growth, and we analyzed the effect of phosphate limitation. Phosphate limitation was chosen because it is a common environmental condition encountered in many marine systems [28]–[30], and it has been described to have a significant effect on primary and secondary metabolism [31], [32]. We report here the astonishing diversity of the exo-metabolome of strain FO-BEG1 and the drastic effect that phosphate limitation has on its composition. These data shed new light onto the complexity of the metabolites secreted by heterotrophic marine bacteria and onto the effect that their metabolic state can have on the composition of DOM in the ocean.

## Materials and Methods

### Growth conditions

Strain FO-BEG1 was cultivated in the carbohydrate/mineral medium (CM) as described by Shieh et al. [33] and modified by Bondarev et al., [23]. For the phosphate surplus condition (+P_i_) phosphate was added to a final concentration of 1.4 mmol L^−1^, whereas no phosphate was added to the phosphate limited (−P_i_) medium. Under −P_i_ conditions the final phosphate concentration was 0.1 mmol L^−1^, and derived from the buffer used for preparing the vitamin solutions. Erlenmeyer flasks of 250 mL were filled with 100 mL of medium and inoculated with 100 µL of a pre-culture grown under +P_i_ conditions. Cultures were incubated at 28°C in the dark and shaken at 120 rpm. We monitored bacterial growth by means of Optical Density (OD) measured at 600 nm using an Eppendorf BioPhotometer (Eppendorf AG, Hamburg, Germany). The OD_600_ was then correlated with the cell number, determined using a Thoma chamber (Brand GmbH, Wertheim, Germany; data not shown). All experiments were performed and sampled in independent experimental triplicates.

Solid phase extraction of dissolved organic matter (SPE-DOM), dissolved organic carbon (DOC) measurements, and Fourier transform ion cyclotron resonance mass spectrometry (FT-ICR-MS) of DOM

For both −P_i_ and +P_i_ cultures, samples were collected immediately after the inoculation (T0) and in the exponential growth phase (T1). Additionally, samples at the end of the logarithmic phase (T2) and during the stationary phase (T3) were collected for the −P_i_ cultures. One more set of samples was collected also in the stationary phase (T2) of +P_i_ cultures. Cells were removed via centrifugation at 10,000 × *g* for 10 min at 5°C, the supernatant was then filtered into 150 mL combusted glass serum-bottles using Acrodisc 25 mm syringe filters with a 0.2 µm pore size GHP membrane (Pall LifeSciences, Ann Arbor, MI, USA), acidified to pH 2 with 2 mol L^−1^ HCl, and stored at 4 °C until further analyses. We collected the samples from all biological triplicates in both +P_i_ and −P_i_ conditions, with the exception of T0.

DOM of the cell-free supernatants was extracted according to the solid phase extraction of dissolved organic matter (SPE-DOM) method described by Dittmar et al. [34]. The extraction was performed using Bond Elute PPL (Agilent Technologies, Wildbronn, Germany) cartridges with a styrene-divinylbenzene (SDVB) polymer modified with a property surface able to retain also the most polar classes of analytes. DOC content of each extract was analyzed using a Shimadzu TOC-VCPH total organic carbon analyzer (Shimadzu, Kyoto, Japan). The extracted DOM samples were then diluted with a mixture of methanol (MS grade) and ultra-pure water (50:50 v/v) to yield a DOC concentration of 20 mg L^−1^ carbon, filtered using a 0.2 µm pore size PTFE filter (Rotilabo, Carl Roth GmbH, Karlsruhe, Germany), and analyzed with a solariX FT-ICR-MS (Bruker Daltonik GmbH, Bremen, Germany) with a 15.0 Tesla magnet and equipped with an electrospray ionization (ESI) source. To maximize our analytical window, all samples were analyzed on the ESI-FT-ICR-MS in positive and negative ionization mode. We minimize the formation of adducts (and dimers of analyte compounds) by applying a gentle in-source collision-induced dissociation (CID) energy. This breaks apart larger adducts (including dimers), but no covalent bonds. All data were acquired with a time domain size of 4 megawords and with a detection range of *m*/*z* (mass to charge ratio) 150 to 2,000. For each run, 500 broadband scans were accumulated. All the mass spectra acquired under both positive and negative mode were analyzed with the Data Analysis version 4.0 SP4 software package (Bruker Daltonik GmbH).

Calibration of the mass spectra was performed as follows: one replicate of −P_i_ T3 was spiked with 0.05 ppm L-arginine (Sigma-Aldrich, Steinheim, Germany), used for the ESI-negative analyses, or with 0.05 ppm Tuning-mix (Agilent Technologies, Palo Alto, CA, USA), used for the ESI-positive analyses. The resulting mass spectra were calibrated internally with reference mass lists, and molecular formulae were assigned for the remaining peaks in the spectra using the Data Analysis software. For the ESI-negative mode, the molecular formulae were assigned to an elemental composition in the following ranges: C 1–∞, H 1–∞, O 1–∞, N 0–4, S 0–2, P 0–1, and allowing errors lower than 0.2 ppm. A mass list with more than 300 masses in the range 150–800 *m*/*z* was obtained and used to calibrate all other acquired mass spectra. Due to the diversity of the samples, the calibration list was adjusted manually to cover always the full detected mass range with at least 40 calibration points. For the ESI-positive mode, the molecular formulae were assigned to an elemental composition in the following ranges: C 1–∞, H 1–∞, O 1–∞, N 0–4, S 0–2, P 0–2, Na 0–1, allowing errors lower than 0.2 ppm. A mass list containing 25 masses covering the range 295–850 *m*/*z* was used to calibrate the other acquired mass spectra, using at least 5 calibration points. All linear calibrations resulted in an average mass error of below 0.05 ppm. Additionally, the instrument was externally calibrated with an in-house marine deep sea DOM reference sample (mass accuracy of less than 0.1 ppm). Before each sample set, blank checks with methanol/ultrapure water 1:1 were measured.

### Sample comparison, molecular formulae assignment, filtration of the datasets, and statistical analysis

Comparison of the mass spectra and isotope (^13^C) identification were performed for the data obtained from both ionization modes, whereas the formulae assignment was done only for the ESI-negative mode. Sodium adducts frequently occur in ESI-positive mode and are considered in our molecular formulae assignment routine. However, other adducts, such as NH_4_^+^, are not easily identifiable, because those cannot be distinguished from other compounds where the same combination of elements is covalently bound. Such a distinction would require extensive additional analyses, such as fragmentation experiments (MS/MS) in the FT-ICR-MS. This was beyond the scope of this study. Because of the inherent uncertainty, we restricted our main analysis to ESI-negative data. With our ESI settings, the ionization in negative mode is highly reproducible and due to loss of H^+^. Other possible adducts (e.g. Cl^−^) can be identified by their unique isotope patterns but were not present in our mass spectra. The computational procedures were performed using an in-house Matlab routine developed by the Max Planck Research Group for Marine Geochemistry. The molecular formulae were assigned in the elemental composition in the following ranges: C 1–40, H 1–∞, O 1–∞, N 0–4, S 0–2, P 0–1, no Na, Fe, Cl and allowing a mass error of maximum 500 ppb. Only peaks with signal to noise ratio > 4 were considered and only formulae with a minimum H/C ration of 0.3 and a maximum O/C ratio of 1 were accepted. All detected ions were singly charged, as indicated by the mass difference between isotopologues (of ^12^C versus ^13^C). Therefore, all detected *m*/*z* values were equivalent to molecular masses.

The ion intensities of the *m*/*z* detected in both ionization modes were normalized by dividing the intensity of each mass by the sum of the 500 highest intensities measured in the respective mass spectra. This normalization procedure was performed independently for each measurement. The normalization was performed after removing all singlets, i.e. masses detected only in one sample out of the seventeen analyzed. In order to have an overview of the similarity among the samples, we performed a non-metric multidimensional scaling (NMDS) for the datasets obtained in both ionization modes, using the Bray-Curtis similarity index for the calculation of the distance matrices. Minimum-spanning trees between all samples were constructed to visualize pairwise sample similarities. Nearest neighbors, i.e. the most similar samples, were identified and graphically connected.

In order to reduce contingent noise and to consider only the molecules produced by the bacteria, we further filtered both datasets using the following criteria: we removed all masses detected in the samples −P_i_ and +P_i_ T0 that did not at least double their normalized ion intensity during the experiment; we removed all masses that were not present in at least all triplicates of one condition at a specific time point; we removed all masses that could contain the isotope ^13^C. The filtered datasets were newly analyzed by means of NMDS, but the samples collected at T0 for both growth conditions were not considered, due to the significant alteration in their *m*/*z* composition derived by the filtration of the datasets. A minimum-spanning tree between all samples was newly constructed. In order to verify the statistical reliability of the clustering observed in all NMDS plots, bootstrap analyses with 1000 reiterations were performed on dendrograms constructed for the similarity matrices obtained using the Bray-Curtis index. The paired group algorithm was used for the construction of the dendrograms. NMDSs, the relative stress values, which are a measure that reflects the degree of deviation of NMDS distances from original matrix distances, the minimum-spanning trees, the construction of the dendrograms, the calculation of the cophenetic correlation coefficients, which are a measure of how faithfully the dendrograms preserve the pairwise distances between the data points, and the bootstrap analyses were carried out by means of the PAST program [35]. Subsequently, in order to identify the unique masses present per time point under both conditions and the masses shared among the growth stages, we created Venn diagrams considering only masses present in all triplicates at the respective time points.

The elemental composition and the modified aromaticity index (AI_mod_; [36]) of each molecular formula assigned to the *m*/*z* detected in ESI-negative mode were used to divide them into molecular categories according to criteria modified after Šantl-Temkiv et al. [37]. For this analysis, we excluded all masses for which multiple molecular formulae were obtained. We divided the molecular formulae into the following categories: peptides (if the molecular formula has an H/C ratio between 1.5 and 2, an O/C ratio lower than 0.9 and includes N), sugars (if the molecular formula has an O/C ratio equal or higher than 0.9 and an AI_mod_ lower than 0.5), saturated fatty acids (if the molecular formula has an H/C ratio equal or higher than 2 and an O/C ratio lower or equal to 0.9), unsaturated aliphatic compounds (if the molecular formula has an H/C ratio between 1.5 and 2, an O/C ratio lower than 0.9 and does not contain N), highly unsaturated compounds (if the molecular formula has an AI_mod_ lower than 0.5, an H/C ratio lower than 1.5, and an O/C ratio lower than 0.9), phenols (if the molecular formula has an AI_mod_ equal or higher than 0.5 and less than 12 C atoms), and polyphenols (if the molecular formula has an AI_mod_ equal or higher than 0.5 and 12 or more C atoms). We emphasize that this categorization is not unambiguous, and alternative structures may exist for a given molecular formula. However, this subdivision provides a helpful overview of likely structures behind the identified molecular formulae.

### Metabolite and pathway annotation

The masses detected in all three biological replicates at each time point in ESI-negative mode were putatively annotated (i.e. level 2 of metabolite identification as defined by the Metabolomics Standards Initiative [38]) using the “transformation mapping” approach [39], after correcting the mass values for the H^+^ loss. This method is based on mapping an experimentally-derived empirical formula difference for a pair of peaks to a known empirical formula difference between substrate-product pairs derived from the KEGG database (Kyoto Encyclopedia of Genes and Genomes [40]). To reduce the number of false positive assignments only metabolites that occurred in one of the *Pseudovibrio* sp. FO-BEG1 pathways (KEGG identifier: psf) were selected for annotation (as listed in KEGG on July 2013). Furthermore, we annotated the obtained masses considering the molecules reported in the Human Metabolome Database [41] and Drug Bank [42]. An additional annotation was performed using a sub-set of compounds reported in the Dictionary of Natural Products Online [43] obtained after performing a search based on the word “bacteria” typed in the property field “Type of organism word”. Since in these three databases the pathways for the compounds are not indicated we could not apply the “transformation mapping” approach; therefore, the annotation was based on one to one matches between the detected masses and the masses of the known compounds, allowing always an error of ≤ 1 ppm.

## Results

### Measurement of the DOC released during bacterial growth and FT-ICR-MS analysis

**Figure 1:**
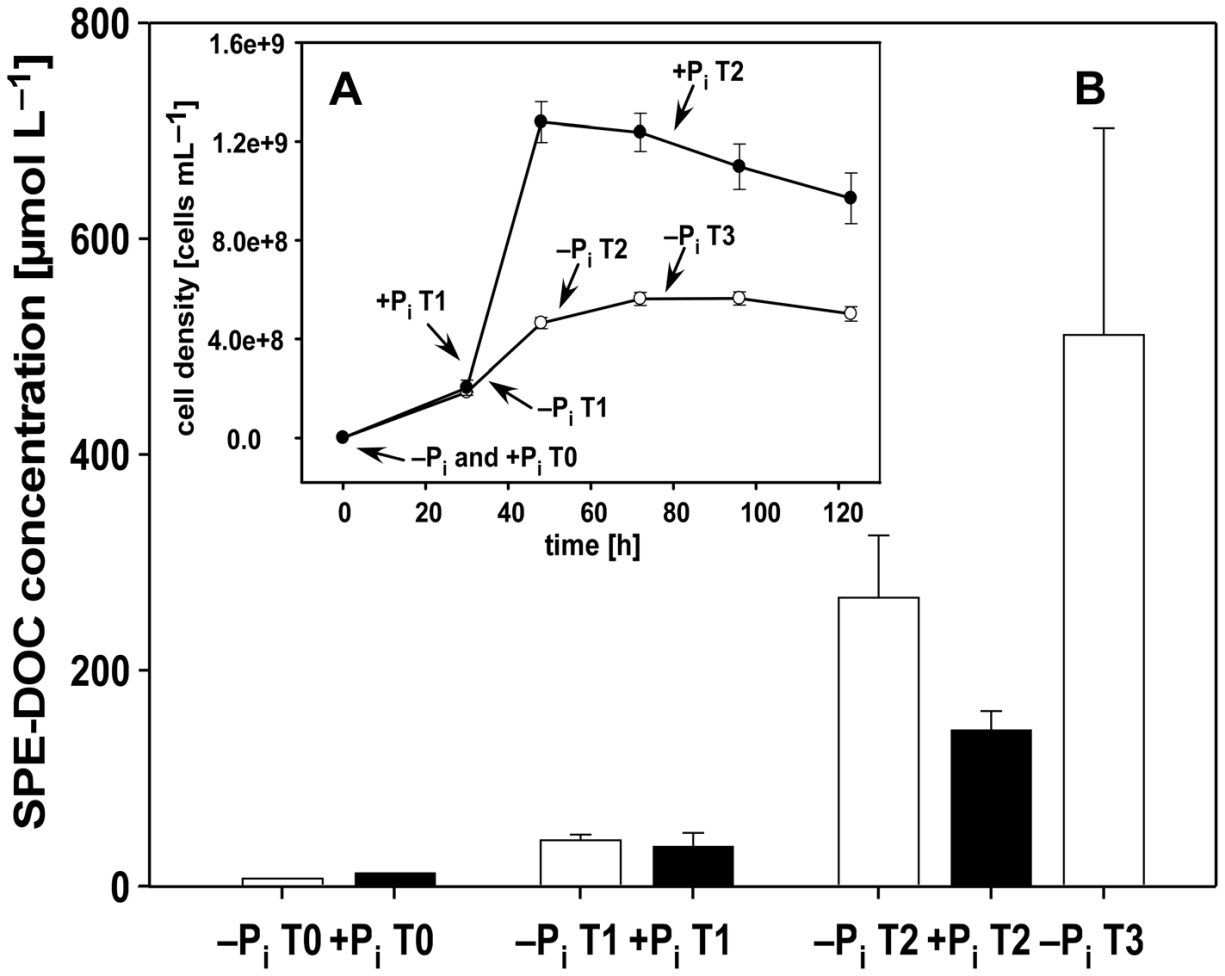
Bacterial growth (A) and concentrations of solid phase extractable dissolved organic carbon (SPE-DOC) (B). The bars (**B**) represent the average concentrations of SPE-DOC measured in the solid phase extracts of the biological triplicates collected during growth under both +P_i_ (black) and −P_i_ conditions (white). The inner panel (**A**) shows the cell growth, measured as cell density over time, for the two tested conditions. Filled circles represent the cultures growing under +P_i_ conditions and empty circles represent the cultures growing under −P_i_ conditions. Error bars indicate the standard deviation of biological triplicates

Phosphate limitation repressed the growth of *Pseudovibrio* sp. FO-BEG1, leading to a final cell density 2.5–3.5 times lower than the one observed under phosphate surplus conditions (Fig. 1A). Under −P_i_ conditions, a slightly higher amount of solid phase extractable dissolved organic carbon (SPE-DOC) was produced during the first half of the exponential phase (T1; Fig. 1B). As observed in T0, the SPE extraction did not retain the provided glucose, which alone would correspond to 60 mmol L^–1^ DOC. Therefore, the measured DOC represented the organic compounds produced and secreted by *Pseudovibrio* sp. FO-BEG1. At T1 under both conditions only around 2 mmol L^−1^ of glucose was taken up by the cells (Romano et al., unpublished data), resulting in a conversion of the initial carbon source in SPE-DOC of 0.4% for −P_i_ cultures and 0.3% for +P_i_ cultures. Despite the lower growth, under −P_i_ conditions the SPE-DOC concentration increased to 267 ± 58 µmol L^−1^ and 511 ± 192 µmol L^−1^ at the end of the logarithmic (T2) and in the middle stationary (T3) phase, respectively. At both growth stages the glucose consumed was around 5 mmol L^−1^ (Romano et al., unpublished data). Consequently, in both cases the SPE-DOC represented 0.9% of the used glucose. Compared to this, the SPE-DOC concentration under +P_i_ conditions during the stationary phase was more than three times lower (144 ± 18 µmol L^−1^; Fig. 1B), representing 0.2% of the consumed glucose.

**Figure 2:**
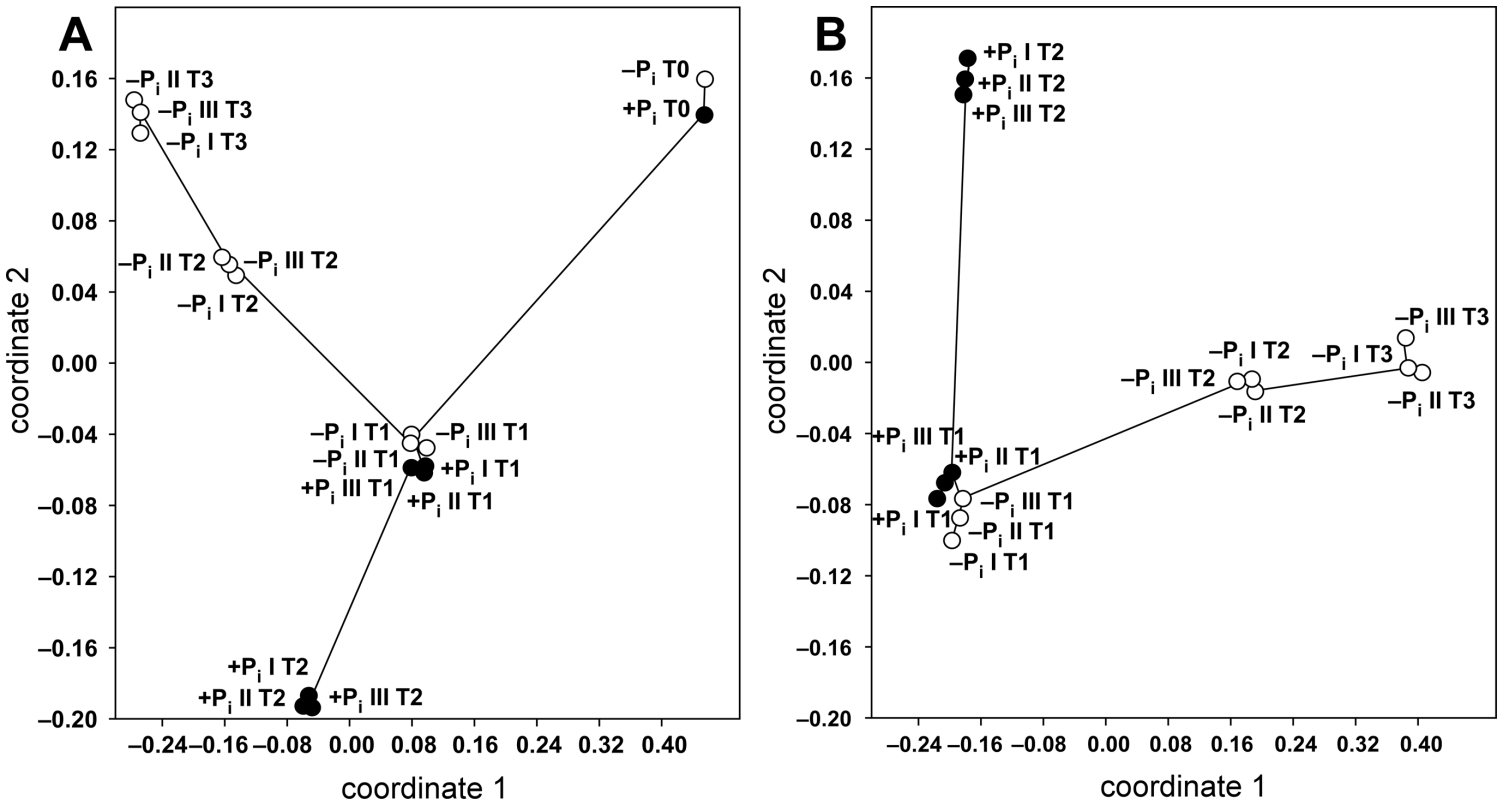
Similarity among the FT-ICR-MS samples analyzed in ESI-negative mode during bacterial growth under +P_i_ and −P_i_ conditions. Non metrical multidimensional scaling (NMDS) was performed by employing the Bray-Curtis similarity index and using the data of the unfiltered (**A**) and filtered (**B**) datasets. All biological triplicates of +P_i_ (filled circles) and −P_i_ (empty circles) conditions are shown. Nearest neighbor samples (i.e. most similar) are connected to visualize pairwise sample similarities. The stress value for both plots is 0.06.

The raw data obtained from the ESI-negative FT-ICR-MS analysis consisted of 23,892 masses ranging from 154 *m*/*z* to 1,930 *m*/*z*. After normalization of the ion intensities, we performed a non-metrical multidimensional scaling (NMDS) in order to evaluate the similarities among the samples (Fig. 2A). As the stress value of the NMDS plot was 0.06, it could be considered a good representation of the calculated distance matrix and thus of the similarity among the samples. The samples collected at T1 for each biological triplicate under both −P_i_ and +P_i_ conditions clustered together and were clearly separated from the samples collected during the rest of the growth period (Fig. 2A). All biological triplicates of the −P_i_ conditions collected at the end of the logarithmic phase and in the stationary phase (T2 and T3) were completely divergent from the samples collected under +P_i_ stationary phase (T2). Moreover, the samples T2 and T3 for the −P_i_ conditions also clustered separately in the plot (Fig. 2A). The bootstrap analysis of the dendrogram constructed for the Bray-Curtis similarity matrix revealed that the divergence among the samples described above was statistically highly significant, since during the 1000 reiterations always the same clustering occurred (Fig. S1A). In ESI-positive mode 17,859 masses were detected, ranging from 153 *m*/*z* to 1,999 *m*/*z*. The NMDS plot obtained for this dataset was characterized by a stress value of 0.07, therefore, it could be considered a good representation of the distance matrix as well (Fig. S2A). All samples had a similar clustering as the one observed in the ESI-negative NMDS plot. One of the main difference was the higher divergence between one −P_i_ replicate (−P_i_ III T1) and the other replicates collected at the same time point. However, the minimum spanning tree showed that this sample shared the highest degree of similarity with the other samples collected under the same growth stage. Additionally, the samples collected at T2 and T3 under −P_i_ conditions showed a higher degree of similarity (Fig. S2A). The bootstrap analysis performed on the respective dendrogram revealed that in > 75% of the cases the samples clustered consistently with the NMDS groups, indicating that the divergences described above were statistically significant (Fig. S3A)

In order to consider only those metabolites that were produced by the strain under the respective conditions, we removed from the datasets all compounds that were already present at T0 and did not at least double their ion intensities during the investigated growth period. Moreover, only compounds present in all biological triplicates at a certain time point and growth condition were further considered. This filtration reduced the ESI-negative dataset to 8,381 masses ranging from an *m*/*z* value of 154 to 998. The NMDS plot (Fig. 2B) performed for this new dataset showed the same clustering pattern as the one constructed for the unreduced dataset (Fig. 2A). 7,499 masses ranging from 163 to 1,234 *m*/*z* were obtained after the filtration of the ESI-positive dataset and, as for the negative mode, the new NMDS plot constructed using these masses showed a clustering consistent with the one of the unreduced dataset (Fig. S2B). The only exception was the higher similarity among the samples +P_i_ T2 and the ones collected at T1 under both phosphate regimes. For both ionization modes, the bootstrap analyses performed on the dendrograms suggested that the clustering of the samples observed in the NMDS plots was statistically significant (Fig. S1B, S3B). Only for the filtered data obtained in ESI-positive mode the divergence among the samples collected at T2 and T3 under phosphate limited condition was not statistically significant, since their divergence occurred in < 50% of the reiterations (Fig. S3B).

**Figure 3:**
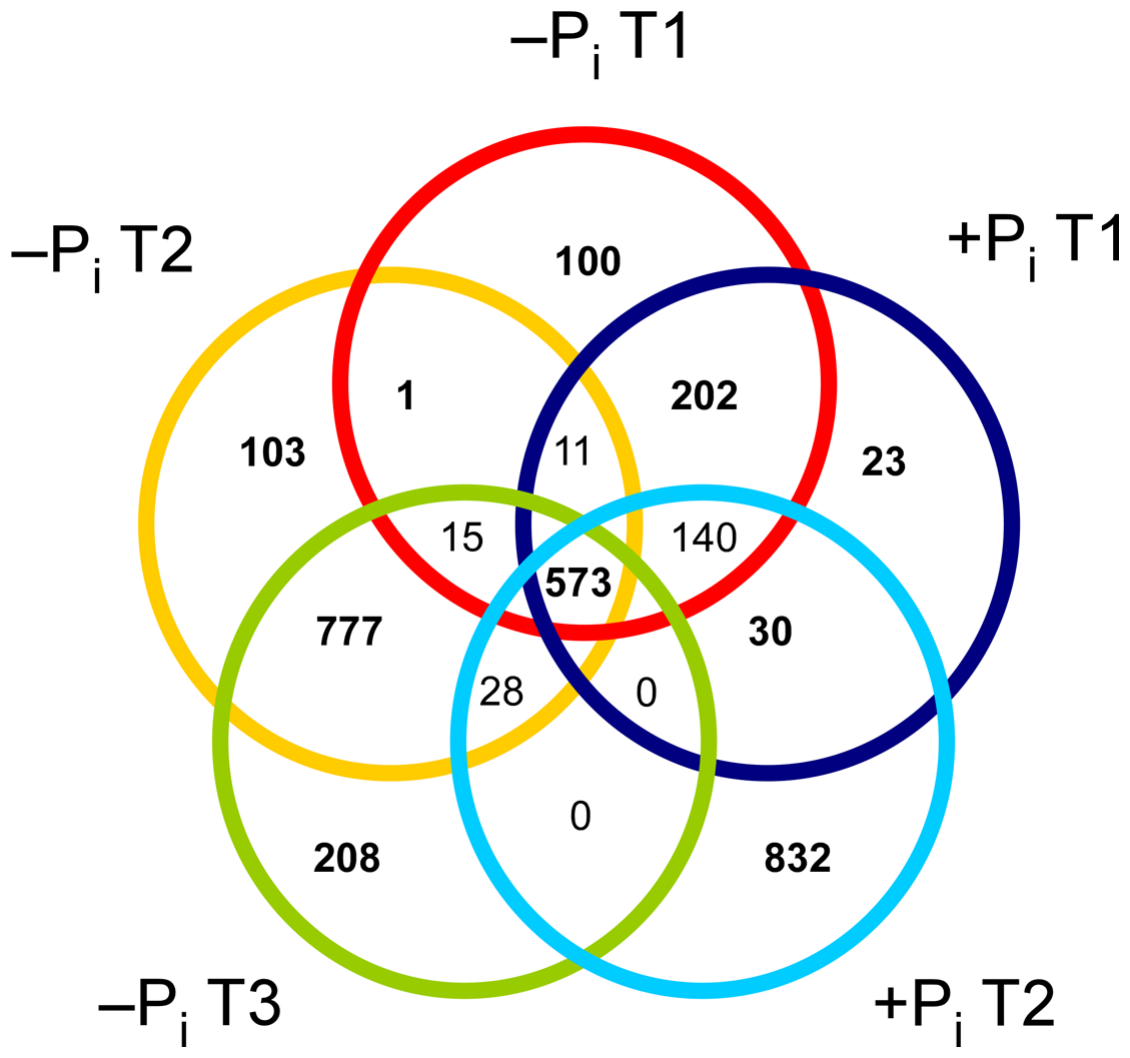
Venn diagram showing unique and shared masses detected in ESI-negative mode in all biological triplicates of the different samples. Only masses detected in all biological triplicates for each time point were considered.

In the Venn diagram constructed considering the masses obtained in ESI-negative mode (Fig. 3), it was evident that the samples collected during the logarithmic growth phase under both +P_i_ and −P_i_ conditions presented 23 and 100 unique masses, respectively. These samples shared 202 masses never detected in the stationary phase. Independent of the condition and the growth phase, we detected 573 masses shared among all samples. The samples collected at the end of the logarithmic and in the stationary phase under −P_i_ conditions (T2 and T3) overall showed 1,088 unique masses never detected in the other time points, whereas in the samples collected in the stationary phase under +P_i_ conditions we detected 832 unique masses (Fig. 3). A highly similar distribution was observed in the Venn diagram obtained for the ESI-positive mode (Fig. S4). The samples collected at the end of the logarithmic phase and in stationary phase under −P_i_ conditions showed a higher number of masses (total of 2,220) than the samples collected in the stationary phase under +P_i_ conditions. In contrast to the results obtained in ESI-negative mode, a higher number of masses (108) was shared among all phosphate limited samples, and a lower number of masses (122) was shared among all samples independent of the condition or growth stage.

The higher variability among replicates observed in ESI-positive mode was likely due to multiple ionization mechanisms, which can result, for example, in ammonium or sodium adduction, both ions present in our culturing medium.

### Conversion of masses obtained in ESI-negative mode into molecular formulae and annotation of metabolites

Of the 8,381 masses detected in ESI-negative mode after the filtration of the dataset described above, we were able to assign molecular formulae to 4,914. Isotopologues were not included in the number of assigned molecular formulae. Of these, 4,122 were unique molecular formulae, i.e. only one molecular formula could be assigned to the respective *m*/*z* value, corresponding to 49% of the *m*/*z* values present in the filtered dataset. A greater percentage of molecular formulae could be assigned to the masses obtained from samples collected at T1 under both +P_i_ and −P_i_ conditions (Table 1). Under +P_i_ conditions an increase in the relative number of formulae containing nitrogen was observed from logarithmic to stationary phase, whereas the percentage of these compounds decreased under −P_i_ conditions (Table 1). Interestingly, during bacterial growth under −P_i_ conditions the relative amount of molecular formulae containing sulfur increased strongly from 45% to 65% of the total assigned formulae (Table 1).

**Table 1:** Overview of the data obtained from the ESI-negative FT-ICR-MS analysis.

	+P_i_ T1	+P_i_ T2	−P_i_ T1	−P_i_ T2	−P_i_ T3
detected masses	2596	4206	3112	3566	2479
unique molecular formulae	1578 (60.8%)	2426 (57.7%)	1931 (62.1%)	1876 (52.6%)	1241 (50.1%)
formulae containing nitrogen	1193 (75.6%)	2085 (85.9%)	1362 (70.5%)	1387 (73.9%)	813 (65.5%)
formulae containing sulfur	648 (41.0%)	1087 (44.8%)	859 (44.5%)	1138 (60.7%)	802 (64.6%)
formulae containing phosphorus	221 (14.0%)	374 (15.4%)	247 (12.8%)	301 (16.0%)	176 (14.2%)

The number of masses detected in all biological triplicates of each time point is shown. The data refer to the dataset obtained after applying the filtration criteria described in the Materials and Methods section. Values in brackets represent the percentages of masses to which a unique molecular formula could be assigned and the percentages of unique molecular formula containing heteroatoms. Isotopologues of assigned molecular formulae are not counted as assigned. Overall, a unique molecular formula could be assigned to 4,122 masses, corresponding to 49% of the obtained *m*/*z*.

**Figure 4:**
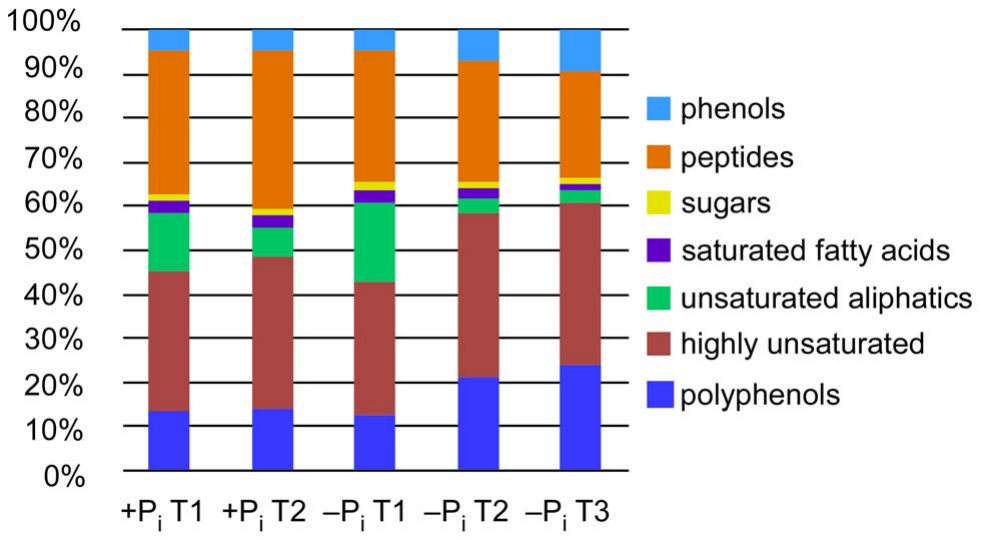
Percentages of molecular formulae attributed to molecular categories. Only masses detected in all biological triplicates for each time point were considered.

After calculating the modified aromaticity index (AI_mod_) we assigned the obtained molecular formulae to specific molecular categories and calculated their relative abundances at different time points (Fig. 4). In agreement with the similarity observed in the NMDS plots, at T1 the composition of the secreted metabolites was similar in both treatments. The major components of the exo-metabolome were compounds with molecular formulae assigned to peptides and highly unsaturated molecules. Only under −P_i_ conditions, a pronounced increase of highly unsaturated, phenolic and polyphenolic compounds and a decrease in peptides and unsaturated aliphatic compounds could be observed during stationary phase.

The ultra-high resolution of the FT-ICR-MS results in precise masses that can be compared and assigned to known compounds present in pathways described for the considered organism and collected in target databases such as KEGG (Kyoto Encyclopedia of Genes and Genomes). The metabolite names reported in the pathways of strain *Pseudovibrio* sp. FO-BEG1 in this database were used to annotate the masses obtained from the FT-ICR-MS analysis. The annotation strategy was based on mapping an experimentally-derived empirical formula difference for a pair of *m*/*z* to an empirical formula difference calculated for substrate-product pairs retrieved from KEGG [39]. It was previously shown that this approach can reduce the false positive rate of putative metabolite annotation by more than fourfold in comparison to searching a compound database using a one to one match approach (peak by peak search), while maintaining a minimal false negative rate [39]. A molecular name could be assigned only to a minor proportion of compounds detected in ESI-negative mode (less than 3%; Dataset S1). For the masses detected in ESI-positive mode, the percentage was even lower. For the reasons outlined above, we did not further consider the ESI-positive results for the annotation of metabolites. We could annotate 85 masses for the sample −P_i_ T1 and of them 55 were assigned to unique metabolites (1.8% of the detected masses in all triplicates). The number of masses assigned to unique metabolites decreased to 46 (65 total annotated masses) for the samples −P_i_ T2 and to 30 for −P_i_ T3 (37 total annotated masses), representing 1.3% and 1.2% of the detected masses in all triplicates, respectively. 49 and 64 masses could be assigned to unique metabolites (73 and 97 total annotated masses) in the samples +P_i_ T1 and +P_i_ T2 (1.8% and 1.5% of the detected masses in all triplicates), respectively. Most of the annotated compounds were intermediates in the metabolism of the amino acids lysine, tyrosine, tryptophan and phenylalanine (Dataset S2 and Fig. S5). In all samples, except −P_i_ T3, several metabolites were also annotated in the pathways of the purine metabolism (Dataset S2 and Fig. S5).

In order to identify possible molecules of biotechnological relevance, we performed three additional annotations using the masses obtained in ESI-negative mode and targeting the Drug Bank (Dataset S3), the Human Metabolome Database (HMDB; Dataset S4), and the Dictionary of Natural Products (DNP; Dataset S5). When the Drug Bank was chosen as target, the maximum number of annotated masses was 327 and was obtained for the sample +P_i_ T2. The same sample presented the highest number of annotate masses (211) also in the annotation performed targeting the DNP. Whereas, using the HMDB, 186 was the maximum number of masses annotated, and it was obtained for the data of the sample −P_i_ T1. In these databases the bio-synthetic pathways are not reported; therefore, the “transformation mapping” approach could not be applied. Consequently, the data obtained have to be considered with caution since a high number of false positive annotations can occur. The annotation performed using the HMDB was in line with the results obtained using the KEGG database, showing mostly intermediate metabolites of amino acid and nucleotide metabolisms (Dataset S4). Although the annotation performed using the Drug Bank database resulted in a higher number of assignments, in every sample several *m*/*z* were annotated as plant metabolites (e.g. epigallocatechin, commonly found in tea leaves; ginkgolide-A, produced by *Ginkgo biloba*) or compounds of synthetic origin (e.g. ibuprofen; Dataset S3). This suggests a high number of false positive annotations; therefore, these data will not be further discussed except when consistent with the other annotation approaches we applied. Finally, the annotation performed using the DNP resulted in the assignment of several masses to compounds having antibacterial, signaling, and enzymatic inhibiting activities (Dataset S5). Interestingly, only in the samples −P_i_ T2 and −P_i_ T3 the *m*/*z* 210.952904 was annotated as tropodithietic acid, which is a potent antibiotic produced by bacteria belonging to the *Roseobacter* clade and the *Pseudovibrio* genus [23], [44].

## Discussion

In order to quantify and characterize the metabolites released by strain *Pseudovibrio* sp. FO-BEG1 into the medium during growth and to evaluate the effect of phosphate limitation on them, we performed an ultra-high resolution mass spectrometry analysis of the bacterial exo-metabolome. Mass spectrometry is the most widely used approach in metabolomic studies [14]. In particular high resolution accurate mass (HRAM) mass spectrometry instruments are receiving progressively more attention, owing to their ability to resolve highly complex samples and to yield accurate mass measurements, which allow precise calculations of the elemental composition [15], [16], [18].

When cells growing under −P_i_ conditions entered stationary phase, they released three times more solid phase extractable dissolved organic carbon (SPE-DOC) than cells growing under +P_i_ conditions (Fig. 1). We are confident that the SPE-DOC concentrations and the number of metabolites obtained are not biased by the presence of compounds derived from the cultivation medium because, as shown by the amount of SPE-DOC at T0, the SPE method did not retain significant quantities of organic compounds present in the medium (Fig. 1B). Moreover, during the filtration of the datasets we removed all *m*/*z* (mass to charge ratio) that were detected at T0 and did not at least double their ion intensities during the experiment. Therefore, all compounds originally present in the medium and not used by the cells during bacterial growth were excluded from the analyses.

It has been known for several years that low phosphate concentrations can induce the production of secondary metabolites ([31] and references therein), which would suggest that under −P_i_ conditions a higher fraction of the carbon source provided was used by *Pseudovibrio* sp. FO-BEG1 for the production of such compounds. In addition, it is known that phosphate limitation can trigger membrane lipid rearrangement, with the substitution of phosphorous-containing with phosphorous-free lipids [45], [46], a phenomenon that we also observed for *Pseudovibrio* sp. FO-BEG1 (Romano et al., unpublished data). Therefore, it is reasonable to hypothesize that due to the membrane rearrangement more cytosolic metabolites could leak out from the cells, explaining the higher production of SPE-DOC under −P_i_ conditions. Consistently, nutrient leakage was also described in a marine yeast strain growing under phosphate limited conditions [47]. Other studies showed that bacteria can convert from 5 to 15% of the provided carbon into DOC [48]–[50], which is one order of magnitude higher than observed in our experiments. However, a precise comparison is difficult because in all mentioned examples different medium composition, growth parameters, and analytic procedures were used.

Rosselló-Móra et al. [19] and Antón et al. [22] analyzed the endo- and the exo-metabolome of different *Salinibacter ruber* isolates during the classification of different ecotypes, and reported that the isolates can be distinguished by their metabolic profiles. Moreover, Brito-Echeverria et al. [20] analyzed the endo- and exo-metabolome of different *Salinibacter* strains in response to different stress conditions, and reported that the exo-metabolome was affected to a greater extent than the endo-metabolome. In all studies, the analyses were performed via FT-ICR-MS, and they are the first reports that provide information about the complexity of the bacterial exo-metabolome. In line with these observations, our analysis revealed that *Pseudovibrio* sp. FO-BEG1 produced and released at least many hundreds of compounds into the medium, and that the composition of this DOC was greatly affected by phosphate limitation. In this respect, FT-ICR-MS represents an ideal and powerful technique to unravel this complexity. We could clearly show that the exo-metabolome composition differs during different growth phases and between the two tested conditions (Fig. 2, 3, S1, S2, S3, S4). These data are consistent with previous studies, which applying low resolution techniques reported that the metabolites secreted by bacteria can change during different growth phases and in response to environmental stresses [8], [11], [21], [51]. One interesting difference between the two phosphate regimes was the higher amount of compounds containing sulfur detected under −P_i_ conditions (Table 1). This suggests that phosphate limitation also influences the sulfur metabolism of *Pseudovibrio* sp. FO-BEG1, increasing the amount of sulfur released into the environment in the form of DOM.

The presence of unique masses detected only at specific time points under both conditions shows a dynamic cycling of organic compounds. Molecules produced during the beginning of the logarithmic growth phase were then taken up again when cells entered stationary phase. A similar phenomenon was observed in a study that investigated the effect of grazing on the DOC production in a pure culture of *Pseudomonas chlororaphis* [50]. Interestingly, even though for each sampling point and each condition we identified hundreds of unique masses, we also detected 573 and 122 masses in ESI-negative and positive mode, respectively, which were always present in our samples independent of the growth stage or the growth condition (Fig 3, S4). It would be interesting to verify whether this “core” exo-metabolome is affected by other environmental changes or it represents a distinctive “metabolic signature” of the strain.

It has been suggested that the trophic status of the environment affects DOM composition via shaping the ecological processes that are responsible for its production [2]. Productive, nutrient rich regions have significant DOM production directly from photosynthesis, whereas oligotrophic, nutrient poor regions have significant DOM production from grazing processes [52], [53]. This difference was attributed to the complexity of the microbial food web in different environments, with the oligotrophic regions having a more effective microbial loop compared to the classical food web described in the productive regions [54]. Our data suggest that in order to understand DOM composition the effect of the environmental nutrient regimes on bacterial physiology should not be underestimated. As we show, it can greatly affect both the amount and the composition of the produced organic compounds.

Comparing the variation of the metabolome of *Escherichia coli* and *Saccharomyces cerevisiae* in response to carbon and nitrogen limitation, Brauer et al. [10] unexpectedly showed global metabolic trends remarkably conserved among these two distantly related microorganisms. Therefore, in order to verify the presence of shared metabolic responses, which could indicate the presence of highly conserved regulatory schemes, it would be of great interest to compare the variations of the exo-metabolome in response to nutrient limitation among different bacteria. Here we show that the nutrient regime greatly influenced the DOM secreted by *Pseudovibrio* sp. FO-BEG1 into the environment. Therefore, it is reasonable to expect that by extending these kinds of studies to different marine bacteria the influences of microbes on DOM composition in natural environments characterized by particular trophic conditions could be better understood.

The molecular formula assignment allowed us to classify the detected masses in molecular categories, giving a broad overview of the types of compounds released during growth. Under phosphate limitation, we observed a higher production of phenolic and polyphenolic compounds when cells entered stationary phase (Fig. 4). Production of phenol was described for the strains *Pseudovibrio* sp. D323 and L4-8 [55], [56]. The crude extract of the spent medium of the latter strain showed a strong antioxidant activity, which is consistent with our finding that strain FO-BEG1 produces different types of phenols and polyphenols, known for their antioxidant properties [57]. Higher production of these compounds under −P_i_ conditions could be related to the increased oxidative stress that cells growing under phosphate limitation might experience [58]–[60], and which we also inferred for strain FO-BEG1 from the comparison of the protein expression between +P_i_ and −P_i_ conditions (Romano et al., unpublished data).

Some of the detected phenolic and polyphenolic compounds could be, for example, tropone derivates. These molecules are commonly produced by bacteria of the *Roseobacter* clade and can have algaecide and antibacterial activity, as, for example, the potent antibiotic tropodithietic acid (TDA; [61], [62]). Previous experiments using high performance liquid chromatography suggested that a compound with the same retention time and UV-visible spectra as the TDA standard was produced by *Pseudovibrio* sp. FO-BEG1 under −P_i_ conditions when cells entered stationary phase (Romano et al., unpublished data). During the FT-ICR-MS analyses, we identified the *m*/*z* 210.952904 with the molecular formula assigned C_8_H_4_O_3_S_2_, which was, considering also its peculiar isotopic patterns due to the presence of two sulfur atoms per molecule, consistent with being TDA. This compound was detected only under phosphate limitation and its ion intensity increased from T2 to T3. Consistently, when the Dictionary of Natural Products (DNP) was used as target for annotating the detected masses, the previously mentioned *m*/*z* was assigned to TDA, and to thiotropocin and troposulfenin (Dataset S5) which are tautomers of TDA [63] and are known to be produced by *Pseudomonas* spp. [64]. In addition, in the same samples, the *m*/*z* 226,947816 was annotated as hydroxytropodithietic acid, which was suggested to derive from the hydroxylation of TDA [65]. Members of the *Roseobacter* clade produce TDA together with an uncharacterized yellow pigment [44] and consistently also the *Pseudovibrio* cultures growing under −P_i_ conditions developed an intense yellow coloration when entered stationary phase (Romano et al., unpublished data). Altogether, this data support our interpretation that TDA was produced during the stationary phase under −P_i_ conditions.

The annotation using the DNP resulted in the attribution of several masses to molecules previously described in marine bacteria, including members of the *Roseobacter* clade (Datasets S5; e.g. 3-(4-Hydroxy-3-nitrophenyl)propanoic acid, cyclo(glutamylglycylprolyl), cyclo(glutamylglycylserylprolyl), homo-ξ-rhodomycinone). Among those, several masses were annotated as cyclic dipeptides produced by *Roseobacter* strains isolated from marine sponges. Cyclic dipeptides are molecules with antibacterial properties and biological and pharmacological effects on cells of higher organisms. It was suggested that they could play a role in bacterial and prokaryote-eukaryote communication [66]–[69]. In the last years several cyclic dipeptides have been isolated from marine organisms such as sponges and algae and from many marine prokaryotes, suggesting that the ability to produce these compounds is widespread among marine bacteria [68]. Considering the phylogenetic and physiological similarity between *Roseobacter*- and *Pseudovibrio*-related bacteria, and the recurrent association between both cyclic dipeptides and *Pseudovibrio* with marine sponges [23], [68], it is reasonable to speculate that *Pseudovibrio* sp. FO-BEG1 released such compounds into the medium. This information offers a solid base for further chemical characterization of these compounds, which could represent new molecules of biotechnological interest.

When the KEGG database was used as target, most of the metabolites assigned to the detected masses were compounds involved in the synthesis of mainly aromatic amino acids (e.g. tyrosine, tryptophan, phenylalanine; Dataset S1, S2, Fig. S5) and nucleotides. Consistently, the annotation performed using the HMDB and the Drug Bank also suggested the presence of intermediates of these metabolisms as, for example, the shikimate pathway (e.g. erythrose-4-phosphate, shikimate-3-phosphate; Datasets S3, S4), which is responsible for the biosynthesis of aromatic amino acids. Release of these compounds was also observed in the analysis of the exo-metabolome of other bacterial and yeast strains [70], [71]. In conditions of “overflow metabolism”, i.e. conditions with an excess of carbon or energy source or in the presence of nutrient limitation, intermediates of different metabolic pathways can be released [72]. Recent evidence suggests that this is a common phenomenon in different microorganisms when they are cultivated under conditions of non-inhibited carbon uptake [70]. Aromatic amino acids are key intermediates in the production of aromatic secondary metabolites [73] suggesting that strain FO-BEG1 is potentially producing such compounds, which, however, are of unknown structure. Unlike observed for other microorganisms [70], no masses were annotated as metabolic intermediates of central metabolic pathways such as the tricarboxylic acid cycle. The main reason for this is that most of these metabolites have a low molecular mass, e.g. fumarate 116.07 Da, which fells outside the *m*/*z* range chosen for our analysis (150-2,000 *m*/*z*).

Among the identified compounds, a smaller number of metabolites could be annotated for the samples collected at T3 under −P_i_ conditions. However, the majority of the annotated compounds belonged to the same pathways identified in KEGG for the other samples. Under phosphate limitation, the number of formulae annotated in the pathway “tyrosine metabolism” and “tryptophan metabolism” using KEGG, and the intermediates of the shikimate pathway detected in HMDB and Drug Bank, decreased strongly from T1 to T3, indicating that these metabolites were taken up again by the cells when they entered stationary phase. This uptake of previously released metabolites was likely done to satisfy specific anabolic needs under this growth conditions.

Production and release of amino acids by bacterial communities was also reported by Kawasaki and Benner [49], and these compounds were shown to be important constituents of DOC in some coastal areas [74], [75], environments where also *Pseudovibrio* strains were often isolated [33], [76]. It is worth pointing out that comparing the list of molecular formulae retrieved from our exo-metabolome study with a list of formulae detected in DOM of the deep North Pacific Ocean [77], we found only 83 shared compounds. However, comparing our data with a list of molecular formulae detected in DOM during and after a phytoplankton bloom in the North Sea (Dittmar et al., unpublished data), we detected 729 matches (18% of the masses with unique molecular formulae assigned) and 91% of them were always present in the natural samples, irrespective of the occurrence of the phytoplankton bloom (Dataset S6). This indicates that, at least on a molecular formula level, a large fraction of the detected compounds are indeed part of natural DOM, and their presence does not seem to be directly related to the immediate activity of primary producers. Consistently, also Kujawinski et al. [78] showed that some molecules detected in a pure culture of “*Candidatus* Pelagibacter ubique” were present in open-ocean DOM.

Our approach represents a high-throughput way of performing metabolomic studies, and it was adequate to capture the diversity of the metabolites released by the bacterium into the environment. However, the translation of the analytical information into existent biological knowledge by using the available tools showed two major drawbacks. The first one regards the reliability of the annotation performed using the HMDB, the Drug Bank and the DNP. By using a one to one match approach (peak by peak search), we were able to assign metabolite names to up to 8% of the detected masses. Especially using the first two databases a high number of double assignments and of non-bacterial metabolites were obtained, suggesting a high rate of false positive identifications. These results confirm previous reports that underlined the limit of single match annotation even when a mass error below 1 ppm is adopted [39], [79]. As pointed out previously, these approaches require the application of orthogonal filtration processes, such as isotopic abundance patterns or “transformation mapping”, which can significantly decrease the number of false positive assignments, generating more reliable information which can be integrated into a biological contest [39], [79].

To increase the confidence in the annotation, we applied the “transformation mapping” approach, using the metabolic pathways of *Pseudovibrio* sp. FO-BEG1 reported in KEGG. Applying this method we were able to annotate less than 3% of the detected masses. The main drawback of this strategy is the incompleteness of the databases used, which can reduce the annotation efficiency by overlooking metabolites that are known, but not yet integrated into the database. For instance, even though we have strong evidence that TDA was produced during the stationary phase under −P_i_ conditions, and the annotation performed using the DNP identified one mass consistent with being TDA, we could not identify it during the annotation processes using KEGG. The reason is the absence of the biosynthetic pathway for TDA among the annotated ones in *Pseudovibrio*. Databases such as KEGG are mostly restricted to genome-reconstruction pathways. Wrongly annotated genes and absence of compounds for which the biosynthetic routs have not been completely elucidated yet can decrease the number of identified molecules in metabolomic studies, and limit the capabilities of techniques such as FT-ICR-MS. This underlines the lack of knowledge we have about the biosynthetic ability of marine bacteria and also the necessity to create more comprehensive databases, containing information about both primary and secondary metabolites.

## Conclusions

In this study we investigated in detail the exo-metabolome of a marine heterotrophic bacterium using ultra-high resolution mass spectrometry. Our work shows that HRAM instruments represent promising tools to unravel the complexity of the metabolites secreted from microorganisms. We show that the exo-metabolome is unexpectedly large and diverse, it is characterized by a dynamic recycling of compounds, and it is drastically affected by the physiological state of the strain. Our data clearly illustrate that phosphate limitation triggered a pronounced increase in the secretion of DOC and at the same time greatly affected its composition, leading to an increased production of functionalized phenols and polyphenols. A Part of the molecular formulae discovered in the exo-metabolome was also detected in natural marine DOM. Therefore, future studies on the exo-metabolomes of different strains and DOM from different locations might help to understand to what extent the compounds secreted by heterotrophic bacteria influence the oceanic DOM composition. The discrepancy between the number of measured masses and the number of annotated molecules obtained using different databases underlines the gap in our knowledge concerning the biosynthetic ability of marine bacteria, indicating the necessity of further work directed to chemically characterize the secreted metabolites. However, the integrated metabolic annotation we performed using multiple databases gave us a first glimpse of the composition of the secreted compounds, suggesting that the large bacterial exo-metabolome can represent a “chemical reservoir” for the discovery of new molecules of biotechnological interest. Our data underline the great biosynthetic ability of heterotrophic bacteria and suggest that, using the words of Traxler and Kolter [80], ‘‘*the chemical landscape inhabited and manipulated by bacteria is vastly more complex and sophisticated than previously thought*’’.

## Supporting Information

Figure S1 **Bootstrap analyses performed on the dendrograms obtained using the paired group algorithm and the Bray-Curtis similarity index calculated for the FT-ICR-MS samples analyzed in ESI-negative mode.** Since the cophenetic correlation coefficients were > 90%, the dendrograms can be considered a reliable representation of the similarity matrices. 1000 reiterations were allowed for the bootstrap analyses. Dendrograms were constructed using the data of the unfiltered **(A)** and filtered **(B)** datasets. All biological triplicates of +P_i_ and −P_i_ conditions are shown.
(TIF)

Figure S2 **Similarity among the FT-ICR-MS samples analyzed in ESI-positive mode during bacterial growth under +P_i_ and −P_i_ conditions.** Non metrical multidimensional scaling (NMDS) was performed by employing the Bray-Curtis similarity index and using the data of the unfiltered (**A**) and filtered (**B**) datasets. All biological triplicates of +P_i_ (filled circles) and −P_i_ (empty circles) conditions are shown. Nearest neighbor samples (i.e. most similar) are connected to visualize pairwise sample similarities. The stress value for **A** is 0.07 and for **B** is 0.08.
(TIF)

Figure S3 **Bootstrap analyses performed on the dendrograms obtained using the paired group algorithm and the Bray-Curtis similarity index calculated for the FT-ICR-MS samples analyzed in ESI-positive mode.** Since the cophenetic correlation coefficients were > 95%, the dendrograms can be considered a reliable representation of the similarity matrices. 1000 reiterations were allowed for the bootstrap analyses. Dendrograms were constructed using the data of the unfiltered **(A)** and filtered **(B)** datasets. All biological triplicates of +P_i_ and −P_i_ conditions are shown.
(TIF)

Figure S4 **Venn diagram showing unique and shared masses detected in ESI-positive mode in all biological triplicates of the different samples.** Only masses detected in all biological triplicates for each time point were considered.
(TIF)

Figure S5 **Number of metabolites annotated in the metabolic pathways of** ***Pseudovibrio*** **sp. FO-BEG1 collected in the KEGG database.** The masses obtained from the ESI-negative FT-ICR-MS analysis were annotated using the MI-Pack package. The bars of each color, representing the different time points, indicate the absolute number of metabolites annotated in the respective pathways reported in the KEGG database.
(TIF)

Dataset S1 **Metabolites annotated using as target the metabolic pathways of** ***Pseudovibrio*** **sp. FO-BEG1 reported in the KEGG database.**
(ZIP)

Dataset S2 **Metabolic pathways present in the KEGG database, which contain the annotated metabolites.**
(XLS)

Dataset S3 **Metabolites annotated using the Drug Bank as target.**
(ZIP)

Dataset S4 **Metabolites annotated using the Human Metabolome Database as target.**
(ZIP)

Dataset S5 **Metabolites annotated using the Dictionary of Natural Products as target.**
(XLS)

Dataset S6 **List of molecular formulae shared between the exo-metabolome of** ***Pseudovibrio*** **sp. FO-BEG1 and the North Sea DOM.**
(XLS)
