# Supplementary figures and images for: Exo-Metabolome of Pseudovibrio sp. FO-BEG1 Analyzed by Ultra-High Resolution Mass Spectrometry and the Effect of Phosphate Limitation
Source: PLoS One. 2014 May 2;9(5):e96038. doi: 10.1371/journal.pone.0096038 (PMC4008564; doi:10.1371/journal.pone.0096038)

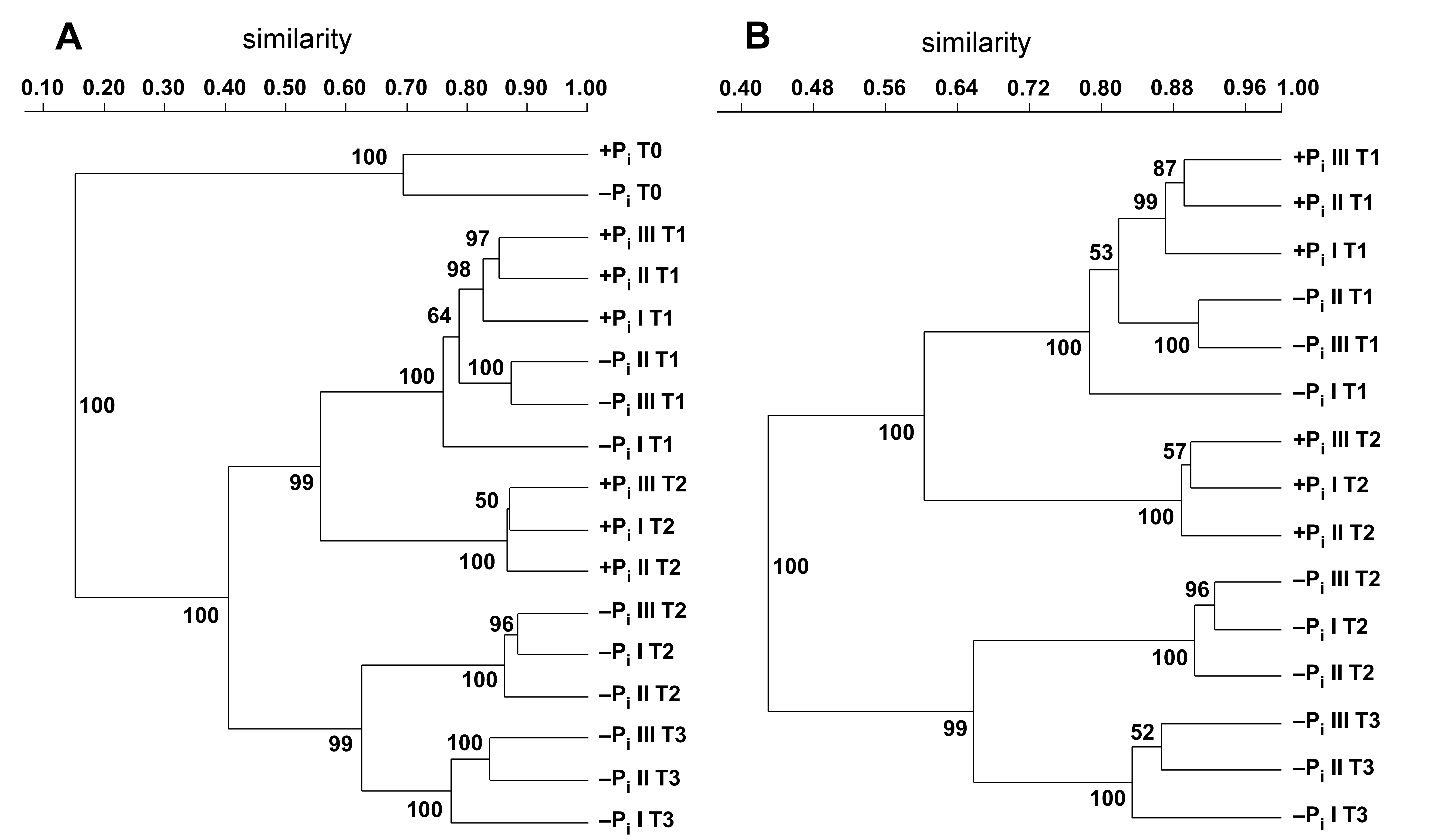

Supplement: Figure S1 — Bootstrap analyses performed on the dendrograms obtained using the paired group algorithm and the Bray-Curtis similarity index calculated for the FT-ICR-MS samples analyzed in ESI-negative mode. Since the cophenetic correlation coefficients were > 90%, the dendrograms can be considered a reliable representation of the similarity matrices. 1000 reiterations were allowed for the bootstrap analyses. Dendrograms were constructed using the data of the unfiltered (A) and filtered (B) datasets. All biological triplicates of +Pi and −Pi conditions are shown. (TIF) [file pone.0096038.s001.tif]

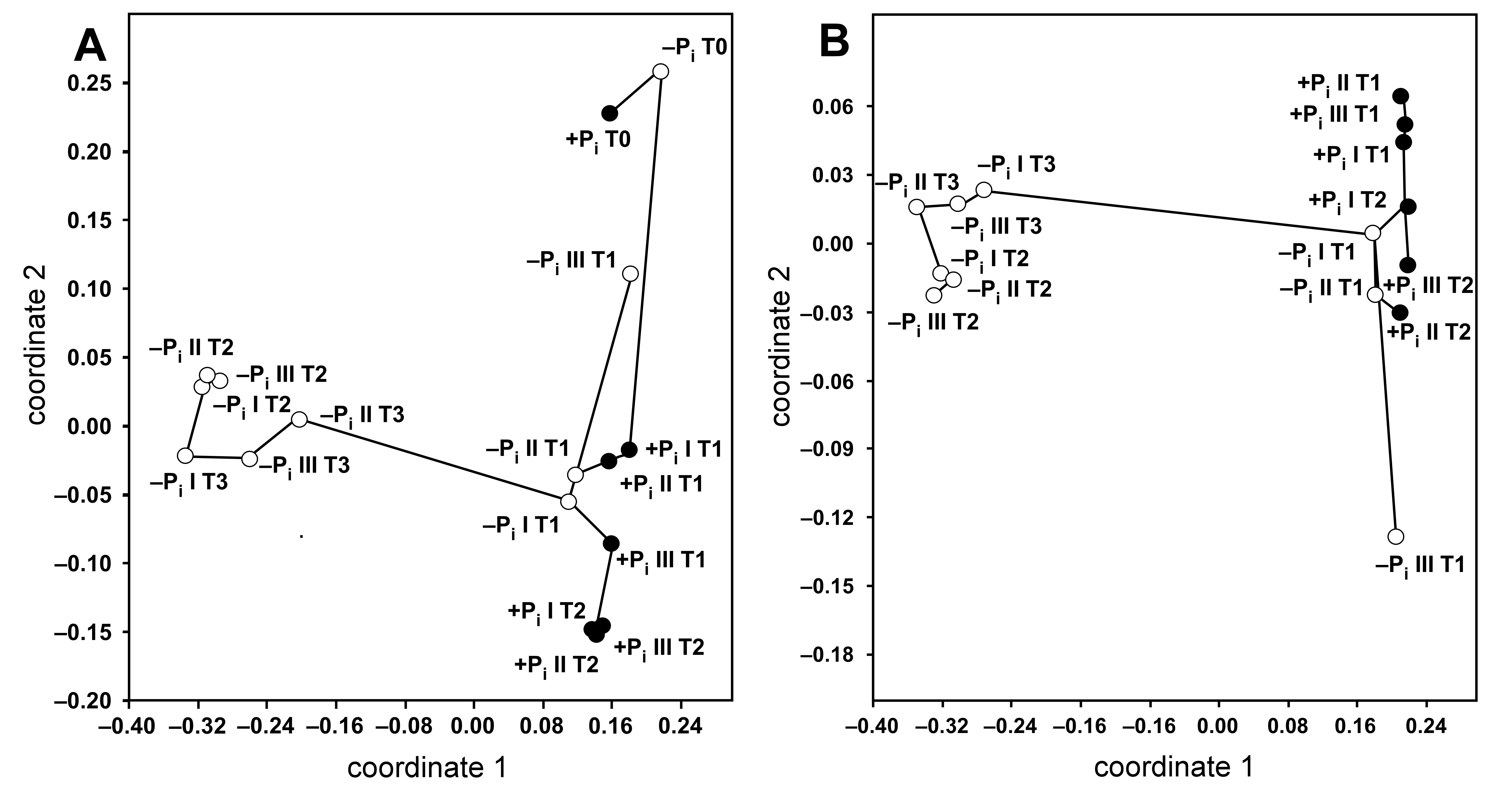

Supplement: Figure S2 — Similarity among the FT-ICR-MS samples analyzed in ESI-positive mode during bacterial growth under +Pi and −Pi conditions. Non metrical multidimensional scaling (NMDS) was performed by employing the Bray-Curtis similarity index and using the data of the unfiltered (A) and filtered (B) datasets. All biological triplicates of +Pi (filled circles) and −Pi (empty circles) conditions are shown. Nearest neighbor samples (i.e. most similar) are connected to visualize pairwise sample similarities. The stress value for A is 0.07 and for B is 0.08. (TIF) [file pone.0096038.s002.tif]

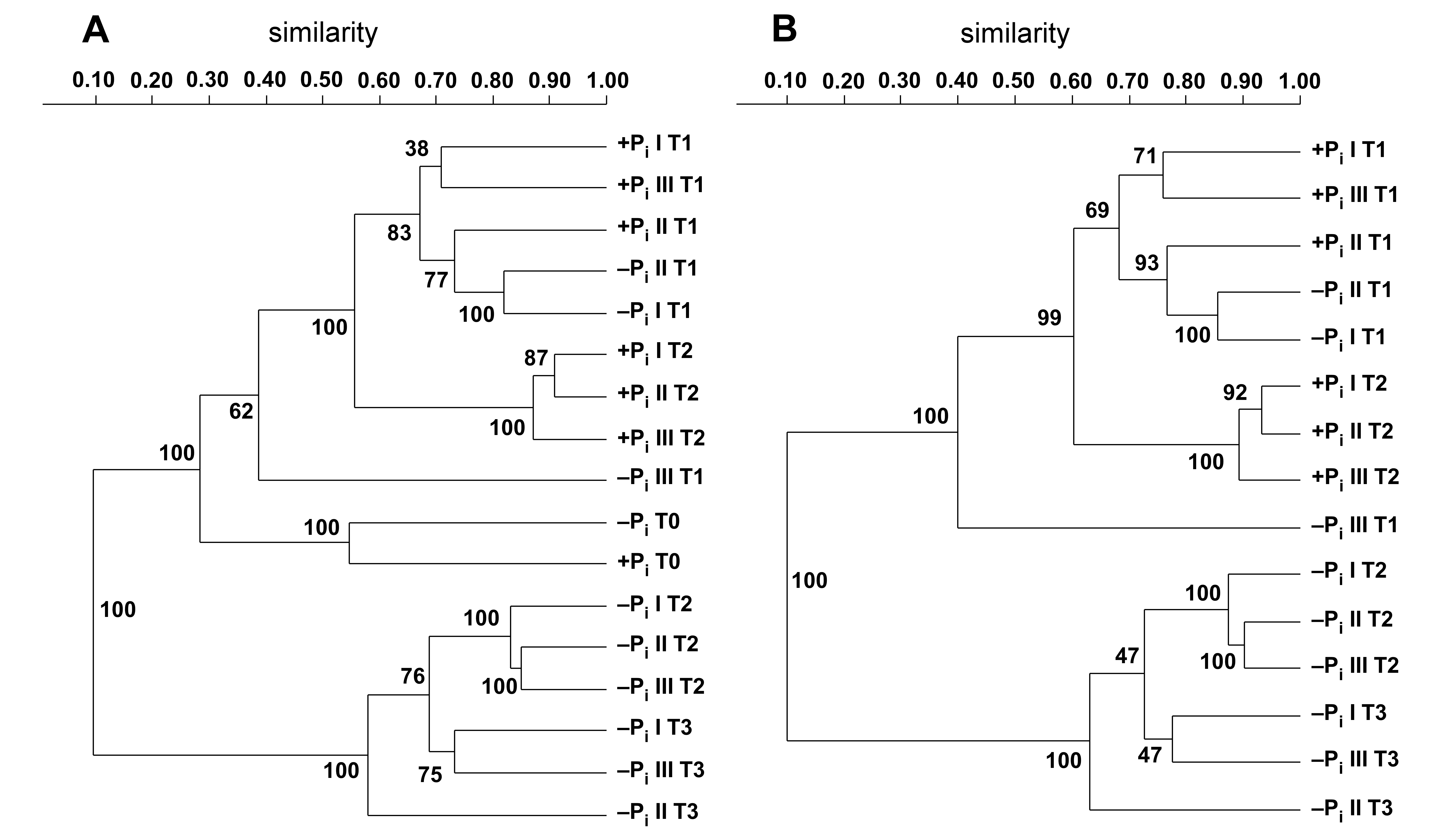

Supplement: Figure S3 — Bootstrap analyses performed on the dendrograms obtained using the paired group algorithm and the Bray-Curtis similarity index calculated for the FT-ICR-MS samples analyzed in ESI-positive mode. Since the cophenetic correlation coefficients were > 95%, the dendrograms can be considered a reliable representation of the similarity matrices. 1000 reiterations were allowed for the bootstrap analyses. Dendrograms were constructed using the data of the unfiltered (A) and filtered (B) datasets. All biological triplicates of +Pi and −Pi conditions are shown. (TIF) [file pone.0096038.s003.tif]

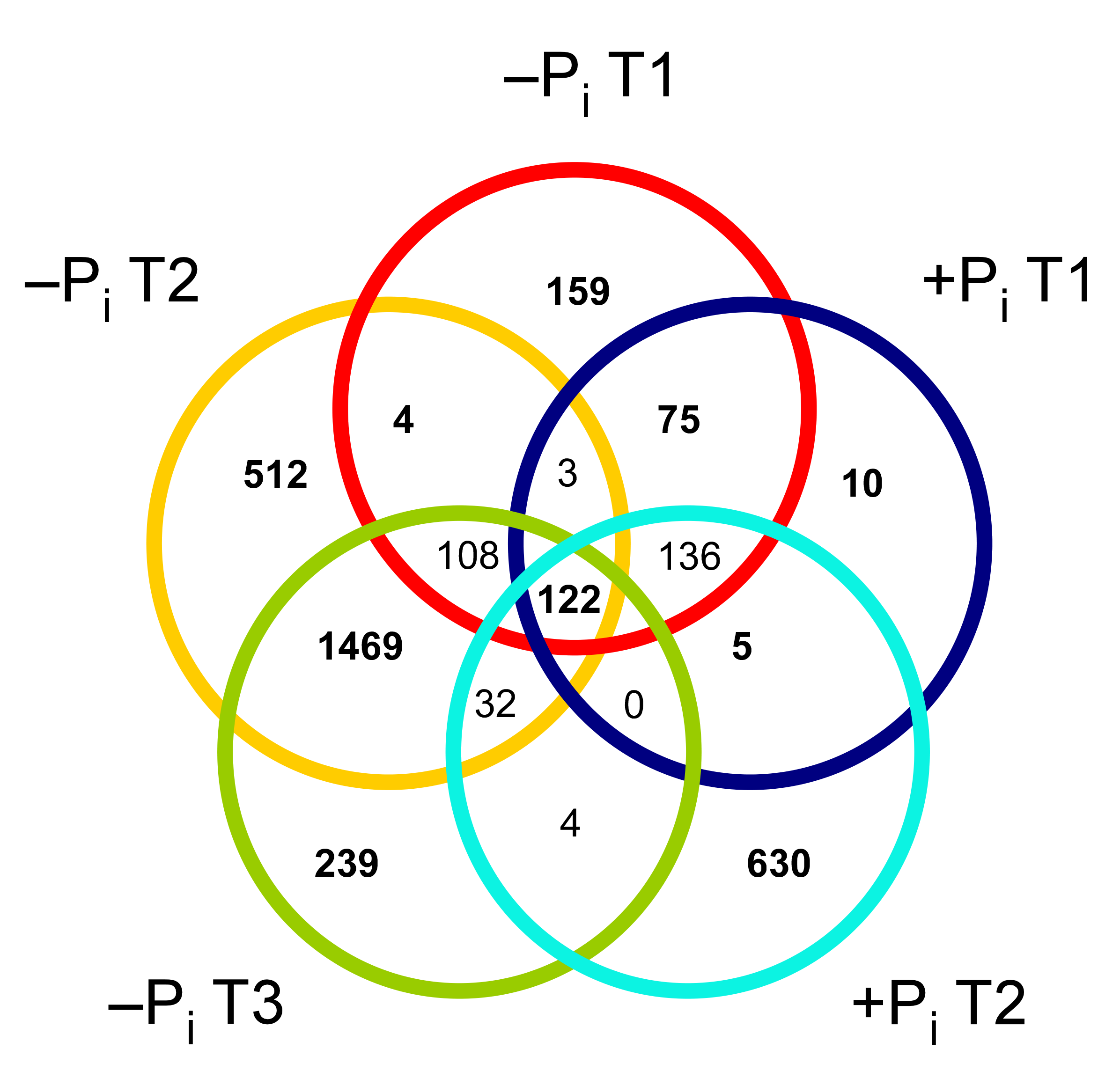

Supplement: Figure S4 — Venn diagram showing unique and shared masses detected in ESI-positive mode in all biological triplicates of the different samples. Only masses detected in all biological triplicates for each time point were considered. (TIF) [file pone.0096038.s004.tif]

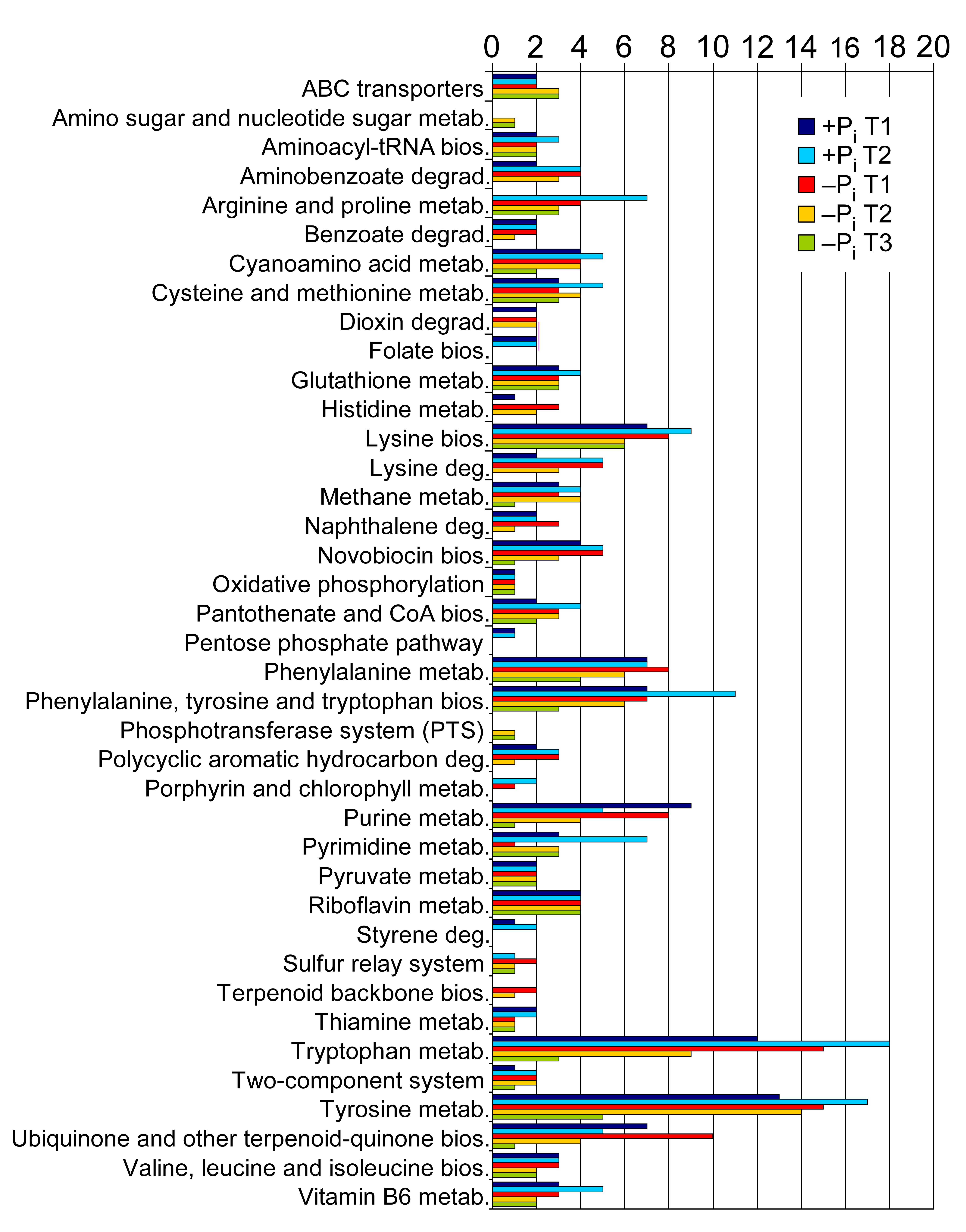

Supplement: Figure S5 — Number of metabolites annotated in the metabolic pathways of Pseudovibrio sp. FO-BEG1 collected in the KEGG database. The masses obtained from the ESI-negative FT-ICR-MS analysis were annotated using the MI-Pack package. The bars of each color, representing the different time points, indicate the absolute number of metabolites annotated in the respective pathways reported in the KEGG database. (TIF) [file pone.0096038.s005.tif]
